# Supplementary material for: Prediction of aneurysmal subarachnoid hemorrhage in comparison with other stroke types using routine care data
Source: PLoS One. 2024 May 31;19(5):e0303868. doi: 10.1371/journal.pone.0303868 (PMC11142441; doi:10.1371/journal.pone.0303868)
Supplement: S1 Table — (PDF) [file pone.0303868.s001.pdf]

S1 Table. Predictor definitions.

Predictor group	Predictor	ICPC	ICD-9	ICD-10	ATC
Cardiovascular system	Cardiac complaints	K01	NULL	NULL	NULL
		K02	NULL	NULL	NULL
	Family history of stroke	NULL	V171	Z823	NULL
	Heart palpitations	K04	NULL	R002	NULL
		K05	NULL	NULL	NULL
	Ankle edema	K07	71907	R600	NULL
		NULL	NULL	R609	NULL
	Cardiac arrhythmia medication	NULL	NULL	NULL	C01B
	Cardiac disease medication	NULL	NULL	NULL	C01A
		NULL	NULL	NULL	C01C
		NULL	NULL	NULL	C01D
		NULL	NULL	NULL	C01E
	Cardiovascular risk management	K49	NULL	NULL	NULL
	Hypertension	K86	401	I10	NULL
		K87	402	I11	NULL

Elevated blood pressure	K85	NULL	NULL	NULL
Antihypertensive medication	NULL	NULL	NULL	C03
	NULL	NULL	NULL	C04
	NULL	NULL	NULL	C05
	NULL	NULL	NULL	C07
	NULL	NULL	NULL	C08
	NULL	NULL	NULL	C09
Antihypertensive medication: atypical	NULL	NULL	NULL	C02
Atrial fibrillation	K78	4273	I48	NULL
Oral anticoagulant drugs	NULL	NULL	NULL	B01AA
	NULL	NULL	NULL	B01AB
	NULL	NULL	NULL	B01AE
	NULL	NULL	NULL	B01AF
Embolism	K93	4151	I26	NULL
	NULL	4449	I82	NULL
Obstetric embolism	NULL	67320	O88	NULL
Heart failure	K77	428	I50	NULL

	NULL	NULL	I110	NULL
Cor pulmonale	K82	416	I27	NULL
Heart murmur	K81	NULL	NULL	NULL
Cardiac atherosclerosis	NULL	4144	I2584	NULL
	NULL	4143	NULL	NULL
Other heart disease	K84	NULL		NULL
	NULL	420	I30	NULL
	NULL	421	I31	NULL
	NULL	422	I32	NULL
	NULL	423	I33	NULL
	NULL	424	I40	NULL
	NULL	425	I41	NULL
	NULL	426	I42	NULL
	NULL	427	I43	NULL
	NULL	429	I51	NULL
	NULL	NULL	I52	NULL
	NULL	NULL	I34	NULL
	NULL	NULL	I35	NULL
	NULL	NULL	I36	NULL

	NULL	NULL	I37	NULL
	NULL	NULL	I38	NULL
	NULL	NULL	I39	NULL
Rheumatic heart disease	NULL	390	I00	NULL
	NULL	398	NULL	NULL
	NULL	39890	I01	NULL
	NULL	NULL	I02	NULL
	NULL	NULL	I03	NULL
	NULL	NULL	I04	NULL
	NULL	NULL	I05	NULL
	NULL	NULL	I06	NULL
	NULL	NULL	I07	NULL
	NULL	NULL	I08	NULL
	NULL	NULL	I09	NULL
Orthostatic hypotension	K88	4580	I951	NULL
Thrombophlebitis	K94	NULL	I80	NULL
Varices	K95	NULL	I86	NULL
	NULL	NULL	I83	NULL
	NULL	NULL	I85	NULL

	Hemorroids	K96	NULL	K64	NULL
	Diabetes mellitus	T90	250	E10	NULL
		NULL	NULL	E11	NULL
	Antidiabetic medication	NULL	NULL	NULL	A10A
		NULL	NULL	NULL	A10B
	Prediabetes	A91.05	7902	R73	NULL
	Hyperlipidemia	T93	272	E78	NULL
	Thrombocyte aggregation inhibitors	NULL	NULL	NULL	B01AC06
		NULL	NULL	NULL	B01AC56
		NULL	NULL	NULL	B01AC08
	Statin use	NULL	NULL	NULL	C10A
		NULL	NULL	NULL	C10B
	Metabolic syndrome	NULL	2779	E88	NULL
Airway	COPD	R95	491	J44	NULL
	Asthma	R96	493	J45	NULL
	Cough suppressants	NULL	NULL	NULL	R05D
	Antihistaminic medication for systemic	NULL	NULL	NULL	R06A

Nasal medication	NULL	NULL	NULL	R01
Medication for obstructive airway disease	NULL	NULL	NULL	R03A
	NULL	NULL	NULL	R03B
	NULL	NULL	NULL	R03C
	NULL	NULL	NULL	R03D
Bronchitis	R78	466	J20	NULL
Allergic rhinitis	R97	NULL	J30	NULL
Upper airway symptoms	R21	NULL	NULL	NULL
	R07	NULL	NULL	NULL
	R22	NULL	NULL	NULL
	R77	NULL	NULL	NULL
Pneumonia	R81	480	J12	NULL
	NULL	481	J13	NULL
	NULL	482	J14	NULL
	NULL	483	J15	NULL
	NULL	484	J16	NULL
	NULL	485	J17	NULL
	NULL	486	J18	NULL

Upper respiratory disease	R74	NULL	J31	NULL
	R75	NULL	J32	NULL
	R76	NULL	J33	NULL
	NULL	NULL	J34	NULL
	NULL	NULL	J35	NULL
	NULL	NULL	J36	NULL
	NULL	NULL	J37	NULL
	NULL	NULL	J38	NULL
	NULL	NULL	J39	NULL
Influenza	R80	487	J09	NULL
	NULL	NULL	J10	NULL
	NULL	NULL	J11	NULL
Hyperventilation	R98	NULL	NULL	NULL
Streptangina	R72	NULL	NULL	NULL
Sarcoidosis	B99.02	NULL	D86	NULL
Pertussis	R71	NULL	A37	NULL
Lung diseases due to external agents	NULL	NULL	J60	NULL
	NULL	NULL	J61	NULL

		NULL	NULL	J62	NULL
		NULL	NULL	J63	NULL
		NULL	NULL	J64	NULL
		NULL	NULL	J65	NULL
		NULL	NULL	J66	NULL
		NULL	NULL	J67	NULL
		NULL	NULL	J68	NULL
		NULL	NULL	J69	NULL
		NULL	NULL	J70	NULL
	Tuberculosis	A70	NULL	A15	NULL
		NULL	NULL	A17	NULL
		NULL	NULL	A18	NULL
		NULL	NULL	A19	NULL
	Emphysema	NULL	NULL	J43	NULL
	Bronchiectasy	NULL	NULL	J47	NULL
	Interstitial lung disease	NULL	516	J84	NULL
Dermatology	Herpes zoster	S70	NULL	B02	NULL
	Dermatitis	S87	NULL	L20	NULL
		S88	NULL	L21	NULL
		NULL	NULL	L23	NULL

	NULL	NULL	L24	NULL
	NULL	NULL	L25	NULL
	NULL	NULL	L26	NULL
	NULL	NULL	L28	NULL
	NULL	NULL	L29	NULL
Psoriasis	S91	6961	L40	NULL
Non-pressure chronic ulcer	S97	NULL	L97	NULL
Dermatological complaints	S03	680	NULL	NULL
	S04	681	NULL	NULL
	S05	682	NULL	NULL
	S18	683	NULL	NULL
	S22	684	NULL	NULL
	S29	685	NULL	NULL
	S74	686	NULL	NULL
	S75	NULL	NULL	NULL
	S79	NULL	NULL	NULL
	S81	NULL	NULL	NULL
	S82	NULL	NULL	NULL

		S84	NULL	NULL	NULL
		S93	NULL	NULL	NULL
		S96	NULL	NULL	NULL
		S98	NULL	NULL	NULL
		S99	NULL	NULL	NULL
		F02	NULL	NULL	NULL
		F03	NULL	NULL	NULL
		F04	NULL	NULL	NULL
		F73	NULL	NULL	NULL
		F99	NULL	NULL	NULL
		D05	NULL	NULL	NULL
ENT	Vertigo	H82	NULL	H80	NULL
		N17	NULL	H81	NULL
		NULL	NULL	H82	NULL
	Hearing problems	H02	NULL	NULL	NULL
		H03	NULL	NULL	NULL
		H77	NULL	NULL	NULL
		H84	NULL	NULL	NULL
		H83	NULL	NULL	NULL
		H85	NULL	NULL	NULL

		Deafness	H86	NULL	NULL	NULL
		Otitis	H71	NULL	H65	NULL
			H72	NULL	H66	NULL
			H74	NULL	H76	NULL
			H70	NULL	NULL	NULL
Gastroenterology		Hepatitis	D72	NULL	B15	NULL
			NULL	NULL	B16	NULL
			NULL	NULL	B17	NULL
			NULL	NULL	B18	NULL
			NULL	NULL	B19	NULL
		Esophagal disease	D84	NULL	K20	NULL
			NULL	NULL	K21	NULL
			NULL	NULL	K22	NULL
			NULL	NULL	K23	NULL
		Appendicitis	D88	NULL	K35	NULL
			NULL	NULL	K36	NULL
			NULL	NULL	K37	NULL
		Diverticulitis	K57	NULL	D92	NULL
		Irritable bowel syndrome	D93	NULL	K58	NULL

Inflammatory bowel disease	D94	NULL	K50	NULL
	NULL	NULL	K51	NULL
	NULL	NULL	K52	NULL
Cholecystitis	D98	NULL	K80	NULL
	NULL	NULL	K81	NULL
	NULL	NULL	K82	NULL
	NULL	NULL	K83	NULL
	NULL	NULL	K84	NULL
Pancreatitis	D99.04	NULL	K85	NULL
Coeliac disease	D99.06	NULL	K900	NULL
Gastric ulcus	D85	NULL	K25	NULL
	D86	NULL	K26	NULL
	NULL	NULL	K27	NULL
	NULL	NULL	K28	NULL
Gastroenterological complaints	D01	NULL	NULL	NULL
	D02	NULL	NULL	NULL
	D03	NULL	NULL	NULL
	D06	NULL	NULL	NULL
	D09	NULL	NULL	NULL

		D10	NULL	NULL	NULL
		D11	NULL	NULL	NULL
		D12	NULL	NULL	NULL
		D16	NULL	NULL	NULL
		D21	NULL	NULL	NULL
		D87	NULL	NULL	NULL
		D78	NULL	NULL	NULL
		D29	NULL	NULL	NULL
		D70	NULL	NULL	NULL
		D18	NULL	NULL	NULL
	Constipation medication	NULL	NULL	NULL	A06A
	Propulsives	NULL	NULL	NULL	A03F
	DRUGS FOR ACID RELATED DISORDERS	NULL	NULL	NULL	A02A
		NULL	NULL	NULL	A02B
Immunology	Rheumatoid arthritis	L88	714	M05	NULL
	HIV/AIDS	B90	042	B20	J05AR
		NULL	V08	Z21	NULL
	Selective immunosup pressants	NULL	NULL	NULL	L04AA

	Antiinflammatory drugs	NULL	NULL	NULL	M01C
		NULL	NULL	NULL	M01A
	Systemic corticosteroid drugs	NULL	NULL	NULL	H02A
		NULL	NULL	NULL	H02B
Lifestyle	Folate deficiency anemia	B81	NULL	D52	NULL
	Iron deficiency anemia	B80	NULL	D50	NULL
	Anemia: other causes	B82	NULL	D64	NULL
	Antianemic preparations	NULL	NULL	NULL	B03A
		NULL	NULL	NULL	B03B
		NULL	NULL	NULL	B03X
	Alcohol intake	P15	NULL	F10	NULL
		NULL	NULL	Y90	NULL
	Smoking	P17	3051	Z716	N07BA
		NULL	V1582	Z720	NULL
		NULL	NULL	F17	NULL
	Drug abuse	P19	NULL	F19	NULL
		NULL	NULL	F55	NULL

			NULL	NULL	F11	NULL
			NULL	NULL	F12	NULL
			NULL	NULL	F13	NULL
			NULL	NULL	F14	NULL
			NULL	NULL	F15	NULL
			NULL	NULL	F16	NULL
			NULL	NULL	F18	NULL
		Weight increase	T07	NULL	NULL	NULL
		Weight loss	T08	NULL	NULL	NULL
		Overweight	T82	NULL	E66	NULL
			T83	NULL	NULL	NULL
		Vitamin deficiency	T91	NULL	NULL	NULL
		Personal history of self harm	NULL	NULL	Z915	NULL
Male-specific		Complaints male genital system	Y04	NULL	NULL	NULL
			Y81	NULL	NULL	NULL
			Y13	NULL	NULL	NULL
		Complaints prostate	Y06	NULL	NULL	NULL
			Y85	NULL	NULL	NULL
		Infertility	N46	6069	N469	NULL

		Erectile dysfunction	P08.01	NULL	N52	G04BE
			Y07	NULL	NULL	NULL
Musculoskeletal		Bone fracture	L72	NULL	M484	NULL
			L73	NULL	M495	NULL
			L74	NULL	NULL	NULL
			L75	NULL	M843	NULL
			L76	NULL	M844	NULL
			NULL	NULL	M907	NULL
			NULL	NULL	M966	NULL
			NULL	NULL	S02	NULL
			NULL	NULL	S12	NULL
			NULL	NULL	S22	NULL
			NULL	NULL	S32	NULL
			NULL	NULL	S42	NULL
			NULL	NULL	S52	NULL
			NULL	NULL	S62	NULL
			NULL	NULL	S72	NULL
			NULL	NULL	S82	NULL
			NULL	NULL	S92	NULL
			NULL	NULL	T02	NULL

	NULL	NULL	T08	NULL
	NULL	NULL	T10	NULL
	NULL	NULL	T12	NULL
	NULL	NULL	T142	NULL
Osteoporosis	L95	NULL	M80	M05BA
	NULL	NULL	M81	NULL
Arthritis	L84	NULL	M15	NULL
	L89	NULL	M16	NULL
	L90	NULL	M17	NULL
	L91	NULL	M18	NULL
	NULL	NULL	M19	NULL
Gout	T92	NULL	M1A	NULL
	NULL	NULL	M10	NULL
Movement related complaints	L01	NULL	NULL	NULL
	L03	NULL	NULL	NULL
	L99	NULL	NULL	NULL
	L92	NULL	NULL	NULL
	L15	NULL	NULL	NULL
	L08	NULL	NULL	NULL

L17	NULL	NULL	NULL
L81	NULL	NULL	NULL
L78	NULL	NULL	NULL
L19	NULL	NULL	NULL
L98	NULL	NULL	NULL
L80	NULL	NULL	NULL
L79	NULL	NULL	NULL
L10	NULL	NULL	NULL
L83	NULL	NULL	NULL
L93	NULL	NULL	NULL
L05	NULL	NULL	NULL
L86	NULL	NULL	NULL
L14	NULL	NULL	NULL
L12	NULL	NULL	NULL
L04	NULL	NULL	NULL
L02	NULL	NULL	NULL
L77	NULL	NULL	NULL
L13	NULL	NULL	NULL
L11	NULL	NULL	NULL
L09	NULL	NULL	NULL

		L16	NULL	NULL	NULL
		L29	NULL	NULL	NULL
		L07	NULL	NULL	NULL
		L87	NULL	NULL	NULL
		L85	NULL	NULL	NULL
Neurology	Headache	N01	7840	G44	NULL
		N02	NULL	G51	NULL
		N90	NULL	NULL	NULL
		N92	NULL	NULL	NULL
	Migraine	N89	346	G43	N02C
	Migraine without aura	NULL	3461	G430	NULL
		NULL	3462	G434	NULL
		NULL	3463	G435	NULL
		NULL	3464	G436	NULL
		NULL	3466	G437	NULL
		NULL	3467	G438	NULL
		NULL	3468	G439	NULL
		NULL	3469	G43A	NULL
		NULL	NULL	G43B	NULL

	NULL	NULL	G43C	NULL
Migraine with aura	NULL	3460	G431	NULL
	NULL	3465	NULL	NULL
Epilepsia	N88	NULL	G40	N03A
Sensibility disorder	N06	NULL	G25	NULL
Restless legs syndrome	N04	NULL	NULL	NULL
Head injury and concussion	N79	NULL	NULL	NULL
	N80	NULL	NULL	NULL
Polyneuropathy	N94	NULL	G61	NULL
	N05	NULL	G62	NULL
	NULL	NULL	G63	NULL
	NULL	NULL	G64	NULL
Parkinson's disease	N87	NULL	G20	N04
Diseases of myoneural junction and muscle	N99	NULL	G70	NULL
	NULL	NULL	G71	NULL
	NULL	NULL	G72	NULL
	NULL	NULL	G73	NULL
Multiple sclerosis	N86	NULL	G35	NULL

			NULL	NULL	G36	NULL
			NULL	NULL	G37	NULL
		Opioid use	NULL	NULL	NULL	N01A
		Logal analgesics	NULL	NULL	NULL	N01B
		Analgesics: other	NULL	NULL	NULL	N02B
Ophthalmology		Cataract	F92	NULL	H25	NULL
			NULL	NULL	H26	NULL
			NULL	NULL	H28	NULL
		Conjunctivitis	F70	NULL	H10	NULL
		Visual disorders	F83	NULL	NULL	NULL
			F84	NULL	NULL	NULL
			F94	NULL	NULL	NULL
		Glaucoma	F93	NULL	H40	NULL
			NULL	NULL	H42	NULL
		Ophthalmologic medication	NULL	NULL	NULL	S01
			NULL	NULL	NULL	S02
Psychiatry		Sleaplessness	P06	NULL	NULL	NULL
		Memory disorders	P20	NULL	NULL	NULL
		Dementia	P70	NULL	F01	N06D

	NULL	NULL	F02	NULL
	NULL	NULL	F03	NULL
ADD/ADHD	NULL	NULL	NULL	N06B
Anxiety or stress related disorders	P74	300	F40	N05B
	P75	NULL	F41	N05C
	P79	NULL	F42	NULL
	P82	NULL	F43	NULL
	NULL	NULL	F44	NULL
	NULL	NULL	F45	NULL
	NULL	NULL	F48	NULL
Psychotic disorders	P72	298	F20	N05A
	P73	NULL	F21	NULL
	P98	NULL	F22	NULL
	NULL	NULL	F23	NULL
	NULL	NULL	F24	NULL
	NULL	NULL	F25	NULL
	NULL	NULL	F28	NULL
	NULL	NULL	F29	NULL
Psychiatric complaints:	P99	NULL	NULL	NULL

other				
	P02	NULL	NULL	NULL
	P78	NULL	NULL	NULL
	P03	NULL	NULL	NULL
	P29	NULL	NULL	NULL
	P01	NULL	NULL	NULL
	P04	NULL	NULL	NULL
Bipolar or manic disorders	P73.02	296	F30	NULL
	NULL	NULL	F31	NULL
Depressive disorder	P76	311	F32	N06A
	NULL	NULL	F33	NULL
Mood disorder: unspecified	NULL	NULL	F34	NULL
Intellectual disabilities	NULL	NULL	F70	NULL
	NULL	NULL	F71	NULL
	NULL	NULL	F72	NULL
	NULL	NULL	F73	NULL
	NULL	NULL	F78	NULL
	NULL	NULL	F79	NULL
Delirium	P71	2930	F05	NULL

		Personality disorders	P80	301	F60	NULL
			NULL	NULL	F63	NULL
			NULL	NULL	F64	NULL
			NULL	NULL	F65	NULL
			NULL	NULL	F66	NULL
			NULL	NULL	F68	NULL
			NULL	NULL	F69	NULL
Socio-economic factors		Financial problems	Z08	NULL	NULL	NULL
			Z10	NULL	NULL	NULL
			Z01	NULL	NULL	NULL
		Problems related to life difficulty	Z29	NULL	Z73	NULL
			Z05	NULL	Z55	NULL
			Z12	NULL	Z56	NULL
			Z14	NULL	Z57	NULL
			Z16	NULL	Z59	NULL
			Z18	NULL	Z60	NULL
			Z19	NULL	Z62	NULL
			Z20	NULL	Z63	NULL
			Z21	NULL	Z64	NULL

		Z22	NULL	Z65	NULL
		Z25	NULL	NULL	NULL
Family history	Oncological diagnosis	A29.02	NULL	Z80	NULL
		A29.03	NULL	NULL	NULL
		A29.04	NULL	NULL	NULL
	Cardiovascular disease	A29.01	NULL	Z824	NULL
		NULL	NULL	Z823	NULL
	Diabetes mellitus	A29.05	NULL	NULL	NULL
	Hyperlipidemia	A29.06	NULL	Z834	NULL
	Psychiatric disease	NULL	NULL	Z81	NULL
Urology	Miction related problems	U02	NULL	NULL	NULL
		U01	NULL	NULL	NULL
		U05	NULL	NULL	NULL
	Hematuria	U06	NULL	NULL	NULL
	Cystitis & pyelonephritis	U71	NULL	N30	NULL
		U70	NULL	N10	NULL
	Kidney failure	U99	585	N17	NULL
		NULL	NULL	N18	NULL

			NULL	NULL	N19	NULL
		Nephrosis	U88	NULL	N00	NULL
			NULL	NULL	N01	NULL
			NULL	NULL	N02	NULL
			NULL	NULL	N03	NULL
			NULL	NULL	N04	NULL
			NULL	NULL	N05	NULL
			NULL	NULL	N06	NULL
			NULL	NULL	N07	NULL
			NULL	NULL	N08	NULL
		Urolithiasis	U95	NULL	N20	NULL
			NULL	NULL	N21	NULL
			NULL	NULL	N22	NULL
			NULL	NULL	N23	NULL
		Urine incontinence	U04	NULL	N394	NULL
Women-specific factors		Precocious puberty	T99.05	2591	E301	NULL
		Ovarian dysfunction	T99.06	256	E28	NULL
		Gestational diabetes mellitus	W84.02	64800	O249	NULL

	NULL	6488	O244	NULL
Diabetes during pregnancy	NULL	6480	O240	NULL
	NULL	NULL	O241	NULL
	NULL	NULL	O243	NULL
	NULL	NULL	O248	NULL
Poor fetal growth	W84.04	6565	O365	NULL
Pregnancy complicating risk factors	W77	NULL	NULL	NULL
Hemorrhage during pregnancy	NULL	640	O46	NULL
Complications during birth	W92	NULL	NULL	NULL
	NULL	641	O61	NULL
	NULL	642	O62	NULL
	NULL	643	O63	NULL
	NULL	644	O64	NULL
	NULL	645	O65	NULL
	NULL	646	O66	NULL
	NULL	647	O68	NULL
	NULL	648	O69	NULL
	NULL	649	O70	NULL

	NULL	768	O72	NULL
	NULL	NULL	O73	NULL
	NULL	NULL	O74	NULL
	NULL	NULL	O75	NULL
	NULL	NULL	O76	NULL
	NULL	NULL	O77	NULL
Encounter for supervision of normal pregnancy	W78	V22	Z34	NULL
Infertility	W15	628	N97	NULL
Preeclampsia or eclampsia	W81.02	6424	O14	NULL
	W81.03	6425	O15	NULL
	NULL	6426	O11	NULL
	NULL	6427	NULL	NULL
Gestational hypertension	NULL	6423	O13	NULL
Hypertensive disorders during pregnancy	NULL	6420	O16	NULL
	NULL	6421	O10	NULL
	NULL	6422	NULL	NULL

	NULL	6429	NULL	NULL
Risk factors during pregnancy	W84.03	NULL	P05	NULL
	W84.04	NULL	P07	NULL
	W84.05	NULL	P08	NULL
	W84.06	NULL	P09	NULL
	W84.07	NULL	NULL	NULL
	W84.08	NULL	NULL	NULL
Birth	W90	V270	Z370	NULL
	NULL	NULL	Z372	NULL
	NULL	V272	Z373	NULL
	NULL	V273	Z374	NULL
	NULL	V274	Z375	NULL
	NULL	V275	Z376	NULL
	NULL	V276	Z377	NULL
	NULL	V277	Z379	NULL
	NULL	650	O80	NULL
Trauma during birth	NULL	NULL	P10	NULL
	NULL	NULL	P11	NULL
	NULL	NULL	P12	NULL

	NULL	NULL	P13	NULL
	NULL	NULL	P14	NULL
	NULL	NULL	P15	NULL
Preterm birth	NULL	6442	O60	NULL
Stillbirth	W91	7799	P95	NULL
	W93	V271	Z371	NULL
Abortion	W82	632	O01	NULL
	W83	634	O02	NULL
	NULL	633	O04	NULL
	NULL	635	NULL	NULL
	NULL	637	O07	NULL
	NULL	639	O08	NULL
Complaints female reproductive organs	X04	NULL	NULL	NULL
	X11	NULL	NULL	NULL
	X13	NULL	NULL	NULL
	X14	NULL	NULL	NULL
	X15	NULL	NULL	NULL
	X16	NULL	NULL	NULL
	X17	NULL	NULL	NULL

	X18	NULL	NULL	NULL
	X84	NULL	NULL	NULL
	X87	NULL	NULL	NULL
Complaints breasts	X18	NULL	NULL	NULL
	X19	NULL	NULL	NULL
	X20	NULL	NULL	NULL
	X88	NULL	NULL	NULL
	99.02	NULL	NULL	NULL
	99.03	NULL	NULL	NULL
	99.05	NULL	NULL	NULL
	X02	6253	N943	NULL
	X03	NULL	N944	NULL
	X09	NULL	N945	NULL
	X10	NULL	N946	NULL
Irregular menstruation	X05	NULL	NULL	NULL
	X06	NULL	NULL	NULL
	X07	626	N91	NULL
	X08	NULL	N92	NULL
Hysterectomy	NULL	NULL	Z90710	NULL

		NULL	NULL	NULL	NULL
		NULL	NULL	NULL	NULL
		NULL	NULL	NULL	NULL
		NULL	NULL	NULL	NULL
		NULL	NULL	NULL	NULL
		NULL	NULL	NULL	NULL
		NULL	NULL	NULL	NULL
		NULL	NULL	NULL	NULL
		NULL	NULL	NULL	NULL
	Hormonal replacement therapy	NULL	NULL	NULL	G03CA
		NULL	NULL	NULL	G03FA
		NULL	NULL	NULL	G03FB
	Intrauterine device	NULL	NULL	NULL	G02B
	Contracepti ve use	W11	V259	Z920	G02B
		NULL	NULL	Z30	G03A
		NULL	NULL	NULL	G03HB
		NULL	NULL	NULL	G03AA
		NULL	NULL	NULL	G03AB
Oncology	Ongological diagnosis	A79	140	C00	NULL

B72	141	C01	NULL
B73	142	C02	NULL
B74	143	C03	NULL
D74	144	C04	NULL
D75	145	C05	NULL
D76	146	C06	NULL
D77	147	C07	NULL
D71	148	C08	NULL
N74	149	C09	NULL
R84	150	C10	NULL
R85	151	C11	NULL
S77	152	C12	NULL
T71	153	C13	NULL
U75	154	C14	NULL
U76	155	C15	NULL
U77	156	C16	NULL
W72	157	C17	NULL
X75	158	C18	NULL
X76	159	C19	NULL
X77	160	C20	NULL

Y77	161	C21	NULL
Y78	162	C22	NULL
NULL	163	C23	NULL
NULL	164	C24	NULL
NULL	165	C25	NULL
NULL	166	C26	NULL
NULL	167	C30	NULL
NULL	168	C31	NULL
NULL	169	C32	NULL
NULL	170	C33	NULL
NULL	171	C34	NULL
NULL	172	C35	NULL
NULL	173	C36	NULL
NULL	174	C37	NULL
NULL	175	C38	NULL
NULL	176	C39	NULL
NULL	177	C40	NULL
NULL	178	C41	NULL
NULL	179	C43	NULL
NULL	180	C44	NULL

NULL	181	C45	NULL
NULL	182	C46	NULL
NULL	183	C47	NULL
NULL	184	C48	NULL
NULL	185	C49	NULL
NULL	186	C50	NULL
NULL	187	C51	NULL
NULL	188	C52	NULL
NULL	189	C53	NULL
NULL	190	C54	NULL
NULL	191	C55	NULL
NULL	192	C56	NULL
NULL	193	C57	NULL
NULL	194	C58	NULL
NULL	195	C60	NULL
NULL	196	C61	NULL
NULL	197	C62	NULL
NULL	198	C63	NULL
NULL	199	C64	NULL
NULL	200	C65	NULL

NULL	201	C66	NULL
NULL	202	C67	NULL
NULL	203	C68	NULL
NULL	204	C69	NULL
NULL	205	C70	NULL
NULL	206	C71	NULL
NULL	207	C72	NULL
NULL	208	C73	NULL
NULL	209	C74	NULL
NULL	230	C75	NULL
NULL	231	C76	NULL
NULL	232	C77	NULL
NULL	233	C78	NULL
NULL	234	C79	NULL
NULL	235	C80	NULL
NULL	236	C7A	NULL
NULL	237	C7B	NULL
NULL	238	C81	NULL
NULL	239	C82	NULL
NULL	NULL	C83	NULL

		NULL	NULL	C84	NULL
		NULL	NULL	C85	NULL
		NULL	NULL	C86	NULL
		NULL	NULL	C87	NULL
		NULL	NULL	C88	NULL
		NULL	NULL	C89	NULL
		NULL	NULL	C90	NULL
		NULL	NULL	C91	NULL
		NULL	NULL	C92	NULL
		NULL	NULL	C93	NULL
		NULL	NULL	C94	NULL
		NULL	NULL	C95	NULL
		NULL	NULL	C96	NULL
		NULL	NULL	Z85	NULL
	Chemotherapy	NULL	NULL	NULL	L01
Other	Minor complaints in primary care	S20	NULL	NULL	NULL
		S95	NULL	NULL	NULL
		S09	NULL	NULL	NULL
		S11	NULL	NULL	NULL

	F16	NULL	NULL	NULL
	F72	NULL	NULL	NULL
	S15	NULL	NULL	NULL
	F76	NULL	NULL	NULL
	F71	NULL	NULL	NULL
	H13	NULL	NULL	NULL
	S10	NULL	NULL	NULL
	S94	NULL	NULL	NULL
	H04	NULL	NULL	NULL
Hypothyroidism	T86	NULL	E01	H03A
	NULL	NULL	E02	H03C
	NULL	NULL	E03	NULL
Hyperthyroidism	T85	NULL	E05	H03B
Fever	A03	NULL	NULL	NULL
Tiredness	A04	NULL	NULL	NULL
	A05	NULL	NULL	NULL
Syncope	A06	NULL	NULL	NULL
Allergy	A12	NULL	NULL	NULL
Wish for euthanesia	A20	NULL	NULL	NULL
Mononuclei	A75	NULL	NULL	NULL

	c infection				
	Trauma, unspecified	A80	NULL	NULL	NULL
	Adverse drug reaction	A85	NULL	NULL	NULL
	Transplanted organ and tissue status	A87.02	NULL	Z94	NULL
	Stoma	A87.01	NULL	NULL	NULL
	Death	A96	NULL	NULL	NULL
Medication use: other	Antibiotic use	NULL	NULL	NULL	J01A
		NULL	NULL	NULL	J01C
		NULL	NULL	NULL	J01D
		NULL	NULL	NULL	J01E
		NULL	NULL	NULL	J01F
		NULL	NULL	NULL	J01G
		NULL	NULL	NULL	J01M
		NULL	NULL	NULL	J01X
	Vaccine	R44	NULL	NULL	NULL
	Mineral supplements	NULL	NULL	NULL	A12A
		NULL	NULL	NULL	A12B
		NULL	NULL	NULL	A12C

	Antiprozoal drugs	NULL	NULL	NULL	P01
		NULL	NULL	NULL	J07BB
	Vitamin supplements	NULL	NULL	NULL	A11A
		NULL	NULL	NULL	A11C
		NULL	NULL	NULL	A11D
		NULL	NULL	NULL	A11E
		NULL	NULL	NULL	A11G
		NULL	NULL	NULL	A11H
		NULL	NULL	NULL	A11J
	Topical antibiotics	NULL	NULL	NULL	D06
	Topical steroid drugs	NULL	NULL	NULL	D07A
		NULL	NULL	NULL	D07B
		NULL	NULL	NULL	D07C
		NULL	NULL	NULL	D07X
	Antifungal medication	NULL	NULL	NULL	D01A
	Antipruritic medication	NULL	NULL	NULL	D04A
	Antiseptic medication	NULL	NULL	NULL	D08
