# Supplementary material for: Prediction of aneurysmal subarachnoid hemorrhage in comparison with other stroke types using routine care data
Source: PLoS One. 2024 May 31;19(5):e0303868. doi: 10.1371/journal.pone.0303868 (PMC11142441; doi:10.1371/journal.pone.0303868)
Supplement: S2 Table — The corresponding coefficients for these predictors in the aneurysmal subarachnoid hemorrhage (aSAH) and intracerebral hemorrhage (ICH) models are also shown. Each coefficient corresponds to the log hazard ratios after applying elastic net penalties. 0 indicates that the predictor is not predictive for that outcome. (PDF) [file pone.0303868.s002.pdf]

S2 Table. All predictors of the acute ischemic stroke (AIS) prediction model. The corresponding coefficients for these predictors in the aneurysmal subarachnoid hemorrhage (aSAH) and intracerebral hemorrhage (ICH) models are also shown.

	aSAH	AIS	ICH
Age	0.163	0.665	0.695
Use of thrombocyte aggregation inhibitors	0.000	0.074	0.031
Comorbidity count	0.000	0.038	0.027
Use of agents acting on the renin–angiotensin system	0.024	0.022	0.039
Use of antidiabetic medication (excl. Insulin)	-0.016	0.013	0.000
Use of beta blockers	0.000	0.008	0.009
Use of statins	0.000	0.007	0.000
HIV/AIDS	0.000	0.005	0.011
Use of calcium channel blockers	0.010	0.005	0.003
Female sex	0.098	-0.018	0.000
Population density	-0.095	-0.073	-0.034

Each coefficient corresponds to the log hazard ratios after applying elastic net penalties. 0 indicates that the predictor is not predictive for that outcome.
