# Supplementary material for: Prediction of aneurysmal subarachnoid hemorrhage in comparison with other stroke types using routine care data
Source: PLoS One. 2024 May 31;19(5):e0303868. doi: 10.1371/journal.pone.0303868 (PMC11142441; doi:10.1371/journal.pone.0303868)
Supplement: S3 Table — The corresponding coefficients for these predictors in the aneurysmal subarachnoid hemorrhage (aSAH) and acute ischemic stroke (AIS) models are also shown. Each coefficient corresponds to the log hazard ratios after applying elastic net penalties. 0 indicates that the predictor is not predictive for that outcome. (PDF) [file pone.0303868.s003.pdf]

S3 Table. All predictors of the intracerebral hemorrhage (ICH) prediction model. The corresponding coefficients for these predictors in the aneurysmal subarachnoid hemorrhage (aSAH) and acute ischemic stroke (AIS) models are also shown.

	aSAH	AIS	ICH
Age	0.163	0.665	0.695
Use of oral anticoagulant drugs	0.000	0.000	0.060
Use of agents acting on the renin–angiotensin system	0.024	0.022	0.039
Use of thrombocyte aggregation inhibitors	0.000	0.074	0.031
Comorbidity count	0.000	0.038	0.027
Other heart diseases	0.012	0.000	0.015
HIV/AIDS	0.000	0.005	0.011
Use of beta blockers	0.000	0.008	0.009
Use of calcium channel blockers	0.010	0.005	0.003
Population density	-0.095	-0.073	-0.034

Each coefficient corresponds to the log hazard ratios after applying elastic net penalties. 0 indicates that the predictor is not predictive for that outcome.
