# Supplementary material for: Prediction of aneurysmal subarachnoid hemorrhage in comparison with other stroke types using routine care data
Source: PLoS One. 2024 May 31;19(5):e0303868. doi: 10.1371/journal.pone.0303868 (PMC11142441; doi:10.1371/journal.pone.0303868)
Supplement: S1 Fig — Each coefficient corresponds to the log hazard ratios after applying elastic net penalties. 0 indicates that the predictor is not predictive for that outcome. (PDF) [file pone.0303868.s004.pdf]

S1 Fig. All predictors of the acute ischemic stroke (AIS) prediction model in relation to the corresponding coefficients of these predictors in the aneurysmal subarachnoid hemorrhage (aSAH) and intracerebral hemorrhage (ICH) prediction models.

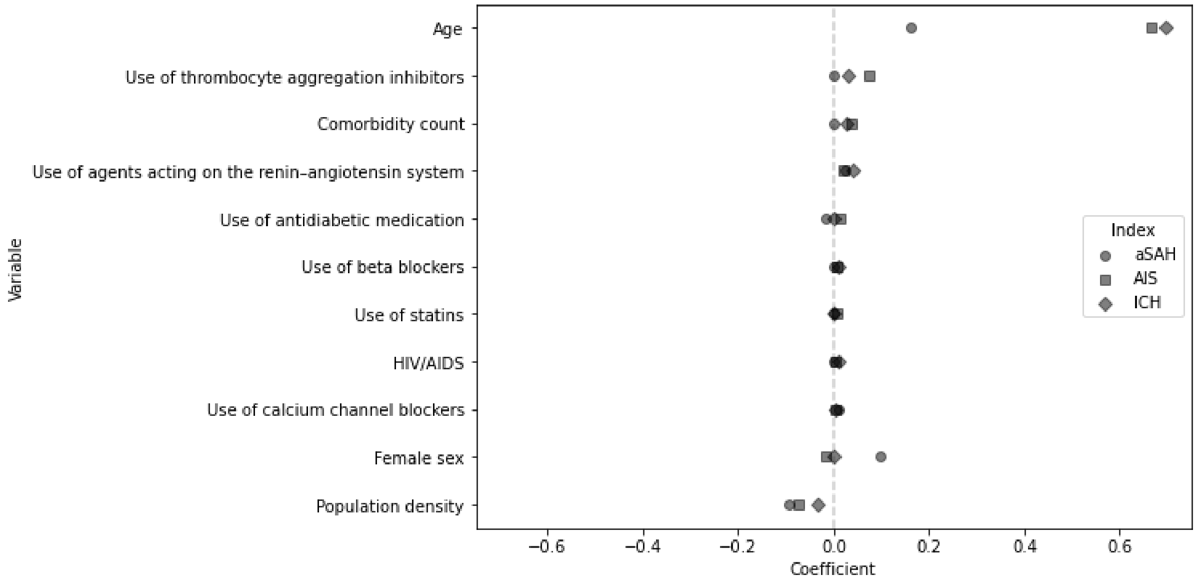

Each coefficient corresponds to the log hazard ratios after applying elastic net penalties. 0 indicates that the predictor is not predictive for that outcome.
