# Supplementary material for: Prediction of aneurysmal subarachnoid hemorrhage in comparison with other stroke types using routine care data
Source: PLoS One. 2024 May 31;19(5):e0303868. doi: 10.1371/journal.pone.0303868 (PMC11142441; doi:10.1371/journal.pone.0303868)

S3 Fig. 10-year Kaplan-Meier curves for observed and predicted survival probability for each outcome.

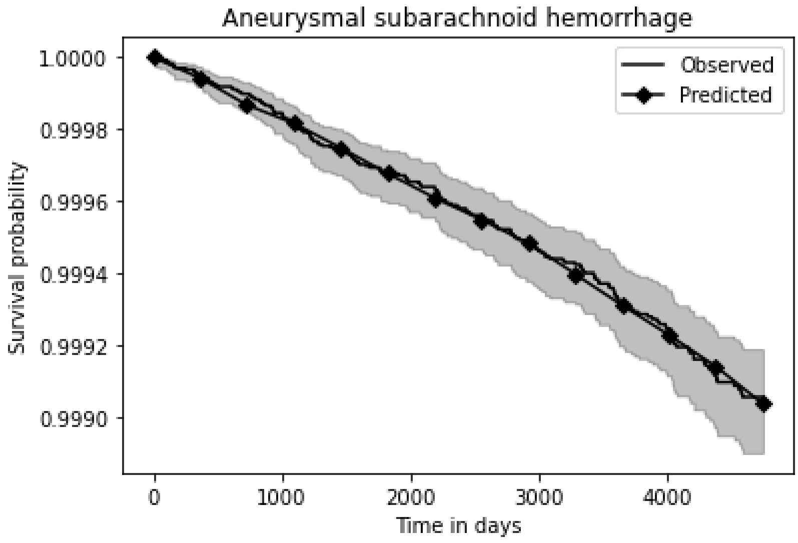

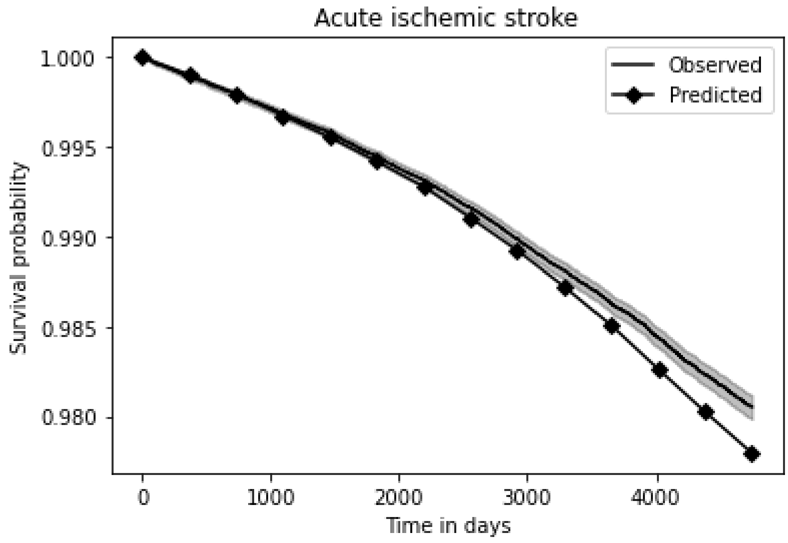

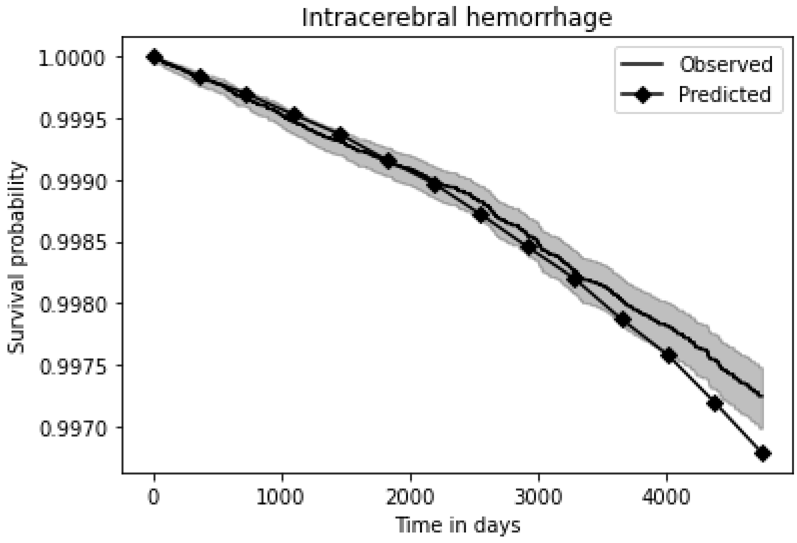

Supplement: S3 Fig — (PDF) [file pone.0303868.s006.pdf]
