# Supplementary material for: Prediction of aneurysmal subarachnoid hemorrhage in comparison with other stroke types using routine care data
Source: PLoS One. 2024 May 31;19(5):e0303868. doi: 10.1371/journal.pone.0303868 (PMC11142441; doi:10.1371/journal.pone.0303868)
Supplement: Attachment — Submitted filename: Response to Reviewers.docx [file pone.0303868.s007.docx]

**Editor comments**

1. Please more clearly enhance the limitations of your prediction model such as limited data related to smoking status and hypertension as well as fulfil all recommended revisions by reviewer 1.

We agree that one of the main limitations of our prediction model concerns the fact that known risk factors for aneurysmal subarachnoid haemorrhage (aSAH) were not systematically assessed for all patients in this study. We have updated the discussion section to make this limitation more explicit (page 15, lines 265-273):

“While increased age correlates with various stroke risk factors [30], it is also correlated~~s~~ with increased opportunities for ~~interaction~~ engagement with healthcare and the possibility of a general practitioner deciding on cardiovascular risk assessment, increasing the likelihood of identifying ~~possible~~ potential stroke risk factors. In our dataset, younger individuals were less likely than older individuals to have available data on known stroke risk factors such as smoking status, alcohol use, blood pressure, and glucose levels [31–35]. As a result, information on these risk factors may not have been available for all patients in our study, potentially lowering model performance.”

And on page 16-17, lines 310-316:

“As a result, the majority of patients in the STIZON cohort lack comprehensive availability of data on medical history. Established aSAH risk factors, such as hypertension and smoking status [32], are thus rarely documented in otherwise healthy individuals [43], making it impossible to determine whether these risk factors are truly absent in the patient, or have not been recorded. This omission may explain why the aSAH prediction model did not include hypertension as a predictor, as well as why the model failed to accurately predict aSAH. ~~Most individuals therefore lack a comprehensive and systematic medical history, e.g. blood pressure is rarely measured in relatively healthy individuals [43]. This lack of systematic data collection may explain why we did not find associations between well-known risk factors (such as hypertension [32]) and the stroke outcomes.~~”

2. Please ensure that your manuscript meets PLOS ONE's style requirements, including those for file naming.

We apologise for the initial oversight. We have updated the text so that it complies with PLOS ONE’s style requirements. In addition, we have renamed the files appropriately.

3. Please state what role the funders took in the study.

This project has received funding from the European Research Council (ERC) under the European Union's Horizon 2020 research and innovation program (grant agreement No. 852173). The funders had no role in study design, data collection and analysis, decision to publish, or preparation of the manuscript.

4. In the online submission form, you indicated that your data is available only on request from a third party. Please note that your Data Availability Statement is currently missing [contact details for the third party, such as an email address or a link to where data requests can be made]. Please update your statement with the missing information.

We apologize for the oversight. We have added STIZON’s contact e-mail and website to the Data Availability Statement.

5. Please upload a new copy of Figure 1 as the detail is not clear.

We have uploaded a new .tiff file with enhanced resolution for Figure 1.

6. Please include captions for your Supporting Information files at the end of your manuscript, and update any in-text citations to match accordingly.

We have added Supporting information captions at the end of our manuscript and updated the text where needed.

7. Please review your reference list to ensure that it is complete and correct. If you have cited papers that have been retracted, please include the rationale for doing so in the manuscript text, or remove these references and replace them with relevant current references.

We have reviewed our reference list and confirm that it is now complete, correct, and that none of the papers referred to have been retracted.

**Reviewer 1**

1. The problem of binary information from billing codes such as smoking status from the corresponding ICD10 code, which does not distinguish between non-smokers and missing data, should be better described

We agree that an exposure definition based on binary ICD or ICPC diagnostic codes does not accurately distinguish between individuals having the exposure and those who have not. We have attempted to make this limitation more explicit and have linked it to the aSAH model’s poorer performance. Please refer to our answer to comment #1 above for the adjustments made.

2. The complex pathology of cerebral aneurysms as a cause of subarachnoid hemorrhage should be presented with regard to genetic and other factors and discussed as a factor for the poorer model prediction compared to ischemic stroke

We agree that the complex pathology of aneurysmal subarachnoid haemorrhage may be an additional reason for the poor performance of the predictive model. We have added this reasoning to the discussion (page 15, lines 273-275):

“Finally, general practitioner data may lack data on risk factors important in the pathogenesis of aSAH, such as a positive family history, genetic risk factors, and yet unknown risk factors.”
